# Study of survival of Holstein cattle of Iran using random regression and single-step BLUP

**DOI:** 10.1016/j.vas.2026.100653

**Published:** 2026-04-10

**Authors:** M. Afrazandeh, J.J. Tourchi, A. Kazemi

**Affiliations:** a Department of Animal Science, Faculty of Agriculture Sciences and Food Industries, Science and Research Branch, Islamic Azad University, Tehran, Iran; b Department of Animal Science, Faculty of Agriculture Sciences, University of Tabriz, Tabriz, Iran; c Animal Breeding Centre, Ministry of Agriculture, Karaj, Iran

**Keywords:** Genomic selection, Productive life, Random regression, Single-step GBLUP, Survival

## Abstract

The survival genetic parameters of Iranian Holstein cattle estimated with the random regression (RR) method in best linear unbiased prediction (BLUP) and single-step genomic BLUP (ssGBLUP) models. The dataset included 30 million test-day records from 1,360,000 first-calving cows in 4,370 herds from 2001 to 2021. Also, the genotypes of 2419 bulls, the frozen semen of which were used for artificial insemination were available. The survival variable for each animal was defined as the number of months from first parturition, with a maximum follow-up period of 100 months thereafter. The cows were assigned to three production groups according to their 305-day milk yield. Results showed the average of survival was 36.2 (±21.9) months. The heritability of the survival trait was 0.049, indicating a relatively small genetic contribution to the variation of the trait. Heritability gradually increased from around 0.02 at the beginning of the period to a peak of 0.06 at approximately 60 months after first calving, and then declined slightly up to 100 months. The RR-ssGBLUP model, with genomic information, significantly improved prediction accuracy (up to 0.74) compared to traditional methods. Higher-producing cows tended to survive longer. These findings highlight the importance of genomic data for enhancing genetic evaluations of survival trait.

## Highlights

• Genetic parameters of survival were estimated up to 100 months of age after first calving.
• Data from 1,360,000 Iranian Holstein cows across 4,370 herds were analyzed.
• Heritability of survival was low (0.049).
• Genomic ssGBLUP models improved prediction accuracy of survival up to 0.74.
• High-producing cows showed greater survival compared to low producers.

## Implications

The results show that using genomic information in random regression model improves the accuracy of genetic evaluation of survival trait in dairy cattle. The high-producing cows tend to stay longer in the herd. The survival mean is comparatively low and research is needed to find out how to improve it.

## Introduction

Survival of animals in Holstein dairy cattle is a trait of major economic and genetic importance. It directly influences herd productivity and profitability by affecting replacement costs and the distribution of cows across productive age groups (Van Pelt et al., 2015). Survival can be defined in several ways: Herd life (HL) refers to the period from birth to culling or death. Productive life (PL) is defined as the time from first calving to culling or death. The term "milking life" refers to the period from first calving to culling or death, excluding any dry periods. Stayability indicates the probability that a cow remains in the herd long enough to raise enough calves to cover her rearing and maintenance costs (Costa et al., 2020).

Survival is a threshold trait expressed as distinct categorical phenotypes, influenced by underlying continuously varying traits (Falconer, 1996). The heritability of survival in dairy cows is generally low, ranging from 0.05 to 0.20 (Caraviello et al., 2004).

The mean of survival of dairy cows is reported to be about 3.0 to 4.5 years (Kerslake et al., 2018), but the maximum production typically occurs during the fifth lactation period, while the greatest profit is usually realized during the sixth lactation period (Horn et al., 2012). Longer survival is economically important for dairy cattle breeders, as it leads to increased profits through lower annual replacement costs and higher average herd yields, achieved by a greater proportion of cows in higher-producing age groups (Van Pelt et al., 2015).

Both intrinsic factors such as milk yield, health, conformation, and reproductive performance (Ferris et al., 2014) and extrinsic factors such as milk price, nutrition, management, policy, feeding costs, and availability of replacement heifers affect survival of animals (De Vries and Marcondes, 2020; Grandl et al., 2016). Livestock survival is a crucial economic factor that significantly impacts the profitability of livestock production (Kern et al., 2016). The survival rate of dairy cows in a herd is determined by the culling rate, which can be either voluntary or involuntary. Involuntary culling occurs due to issues such as reproductive problems, mastitis, and lameness. In contrast, voluntary culling is based on the farmer's decision, often prompted by low milk production, highlighting the importance of including survival in breeding programs (Van Arendonk, 1985). Since involuntary culling negatively affects farm productivity, many countries include the survival trait in their breeding programs and predict the breeding value for this trait.

The random regression (RR) model is commonly used for longitudinal data or repeated records collected throughout an animal's life (Hill and Brotherstone, 1999) and has been applied in survival analysis (Jamrozik et al., 2008). Survival data can be complete (uncensored) or incomplete (censored), with live cows at the time of analysis considered censored (Bewick et al., 2004). In RR models, an individual’s survival from first calving to culling or death is coded as 1 for each month the animal is alive, and as 0 for all months after death or culling until the end of the period, allowing inclusion of censored data in genetic analyses.

Genomic evaluation of young bulls reduces generation intervals and improves the accuracy of genetic predictions. The single-step algorithm integrates phenotypes, genotypes, and pedigrees to predict genomic breeding values, although over-dispersion (inflation) may occur for young bulls without daughters’ phenotypes. This inflation means that genomic breeding values may be overestimated when phenotypic data from daughters are not yet available (Aguilar et al., 2010; Masuda et al., 2018; Tsuruta et al., 2011). Pre-selection and incompatibility between genomic and pedigree matrices can introduce bias, which can be mitigated using scaling factors in combining matrices (Vitezica et al., 2011). Additionally, bias may arise from incompatibility between the pedigree-based relationship matrix (A) and the genomic relationship matrix (G). To achieve compatibility between pedigree and genomic information for genotyped animals, different scaling factors are used to combine the inverse of the genomic relationship matrices ($G^{-1}$) and the pedigree of these animals ($A_{22}^{-1})$.

The aim of this study was to investigate the genetic architecture of survival in Iranian Holstein dairy cattle from first calving up to a maximum of 100 months. The results of BLUP and ssGBLUP models compared to assess the accuracy and dispersion of genetic predictions, with the goal of proposing a suitable model for genomic evaluation of the survival trait.

## Material and methods

### Data of phenotype

The data which were provided by the National Animal Breeding Center of Iran included 30,947,911 test day records from 1,363,534 first-calving cows in 4,370 herds collected from 2001 through 2021 and genotypic information from 2,419 bulls used for artificial insemination were available. Also, the breeding values of 305-day milk yield for first lactation, used to estimate the correlation with breeding values for survival, were obtained from the National Animal Breeding Center for uncensored cows. For the survival trait, the number of months each cow was alive from first calving till culling or death was coded as one; thereafter, a value of zero was assigned until 100 months. For example, if an animal was culled or died 40 months after first calving, the survival for it was assigned 40 ones and 60 zeros. Thus, for each uncensored cow, there were 100 values of 1 and zero. Meanwhile, for censored animals, the number of values were equal to the number of months from the first calving until the last recording.

The data were screened according to the following criteria: 1) Age at first calving between 19 and 40 months; 2) Cows that were censored must have survived at least one month after first calving; 3) Cows that had more than 100 months since first calving were considered in the data analysis only up to the 100th month. 4) Cows with more than 50 months since first calving should have completed at least three lactations. Some studies have suggested applying the survival trait, adjusted for milk production, to account for voluntary culling based on milk yield. (Allaire and Gibson, 1992; Dekkers et al., 1994). The adjustment of survival accounts for voluntary culling due to low milk production. Therefore, according to the 305-day milk yield for first calving, the cows were allocated to three groups as following:

1- G1 - less than 7,600 kg
2- G2 - 7,600-10,000 kg
3- G3 - more than 10,000 kg

The data file was screened to include sires with more than two daughters and in each herd-year season (HYS) of first calving with more than five observations. The final dataset comprised of 45,471,292 test-day observations from 630,945 dairy cows, 6,536 sires, and 1,463 herds (Table 1).

**Table 1:** Summary of dataset and descriptive statistics.

The number of cow	1,363,534
The number of herd	4,370
The number of test day records	30,947,911
The number of genotyped animal	2,419
After data screening	
Number of cows	630,945
Number of cows censored	251,961
Number of cows uncensored	378,984
Censoring rate	0.4
The number of herd	1,463
Number of sire	6,536
Mean of fist calving milk yield (kg)	9,222 (±2,276)
Survival (months)	36.2 (±21.9)
Total number of data created	45,471,292

### Data of genotype

The Genotypic information was available for 2,419 Holstein bulls. Among these, 1,378 bulls were genotyped using medium-density SNP chips (20,000 to 60,000 SNPs), 940 bulls using high-density SNP chips (more than 60,000 SNPs), and 101 bulls using low-density SNP chips (fewer than 20,000 SNPs). Quality control was performed using QCf90 software (Misztal et al., 2002), which removed SNPs with a minor allele frequency (MAF) of less than 0.01, a call rate below 0.95, and those failing the Hardy-Weinberg equilibrium test (p < 10⁻⁶). The reference panel consisted of SNPs overlapping with the 50 k panels. Using FImpute software, SNPs from the higher-density panels that overlapped with the reference panel were imputed to the 50 k panel. Similarly, SNPs from the low-density panels were imputed to the reference panel (Sargolzaei et al., 2014). As a result, all animals had genotypic information for the same set of SNPs (Guarini et al., 2018). Finally, 41,099 SNPs and 2,419 genotyped bulls were included in the analysis.

### Statistical model

#### Random regression BLUP (RR-BLUP)

The genetic parameters of the survival trait were estimated using a Random Regression Animal Model (Model 1) with Restricted Maximum Likelihood (REML). This analysis was conducted using the remlf90 software (Misztal et al., 2002).

(1) $$y_{itm}=Level_{i}+\sum_{k=1}^{n_{f}}\beta_{k}{ɸ}_{k}\left(t\right)+\sum_{k=1}^{n_{a}}a_{km}{ɸ}_{k}\left(t\right)+\sum_{k=1}^{n_{pe}}pe_{km}{ɸ}_{k}\left(t\right)+\sum_{k=1}^{n_{HYS}}HYS_{kl}{ɸ}_{k}\left(t\right)+e_{itm}$$

The $y_{itm}$ is the observation (survival) of cow m at time t in the milk production level group i. $level_{i}$ group is the fixed effect of milk production group level (i=1,2,3). The $\beta_{k}$ is the kth fixed regression coefficient (The number of months after the first calving.), Variables $a_{km}$, $pe_{km}$ and $HYS_{kl}$ are the kth genetic random regression coefficients for cow m, the permanent environmental effect for cow m, and the herd-year-season of first calving, respectively. The ɸ matrix contains standardized Legendre polynomial coefficients for time in the range of -1 to +1. The rows of this matrix correspond to the observation time (t), while the number of columns relates to the degree of the polynomial. Thus, ${ɸ}_{k}\left(t\right)$ is the kth covariate for time t. The $e_{itm}$ is the residual effect of each observation. The $n_{f}$, $n_{a}$, $n_{pe}$ and $n_{hys}$ are the number of covariates to fit the respective regressions: fixed, additive genetic, permanent environment, and HYS. The degree of fit for each level of random effects and fixed regression is evaluated using three covariates (quadratic) and four covariates (cubic), respectively.

The covariance matrices for model RR-BLUP are as follows:$A\;⊗K_{a}=Var\left(a\right)$

$$I\;⊗K_{pe}=Var\left(pe\right)$$

$$I\;⊗K_{HYS}=Var\left(hys\right)$$

$$I\;\sigma_{e}^{2}=Var\left(e\right)$$

A is pedigree matrix. $K_{a}$, $K_{pe}$, and $K_{HYS}$ are covariance matrix between basic coefficients for genetic, permanent environment, and HYS effects. I is identity matrix. The ⊗ is Kroneker product. $\sigma_{e}^{2}$ is random error variance.

#### Random regression ssGBLUP (RR-ssGBLUP)

The ssGBLUP model is like the RR model, except that the H matrix is ​​used instead of the A matrix. In this model, the covariance matrices are the same as in the random model, except for the following:

$$H\;⊗K_{a}=Var\left(a\right)$$

H is a combined matrix (using genomic information from the pedigree).

Using the RR-ssGBLUP model, which substitutes the realized relationship matrix H for the pedigree-based relationship matrix A, the GEBVs were estimated. While the computation of H can be time-intensive, its inverse ($H^{-1}$) has a simple structure, and was calculated as follows (Aguilar et al., 2010).

(2) $$H^{-1}=A^{-1}+\left[\begin{array}{cc} 0 & 0\\ 0 & \tau G^{-1}-\omega A_{22}^{-1} \end{array}\right]$$

Where $G^{-1}$ is the inverse of the genomic relationship matrix (Lourenco et al., 2020; VanRaden, 2008), $A^{-1}$ is the inverse of the relationship matrix, and $A_{22}^{-1}$ is the inverse of section A related to genotyped animals. To make G invertible, the blending parameter of 0.05 was used; which means $G=0.95G_{0}+0.05A_{22}^{-1}$. The $G_{0}$ is genomic matrix before being combined with pedigree information (matrix A). The scaling factors coefficients τ and ω were used to account for reduced genetic variance and varying pedigree depths, respectively, to make $G^{-1}$ compatible with $A_{22}^{-1}\;$and also$\;A^{-1}$. To determine the optimal scaling factors that yield the highest accuracy and lowest dispersion in GEBV survival estimations, different values for τ and ω were evaluated.

### Estimation of genetic parameters

Using the coefficients of the covariance components determined by the RR in Eq. 3, the covariance between observations in different periods was estimated.

(3) $$Cov=ɸK{ɸ}^{\prime }$$

The ɸ is the time matrix, K is the coefficients of the covariance components, and ${ɸ}^{{}^{\prime }}$ is the transpose of the ɸ matrix. The diagonal elements of the covariance matrix derived from Eq. 3 were used as the variances of random effects to estimate heritability, as defined in Eq. 4.

(4) $${\hat{h}}^{2}=\frac{{\hat{\delta }}_{a}^{2}}{{\hat{\delta }}_{PE}^{2}+{\hat{\delta }}_{HYS}^{2}+{\hat{\delta }}_{a}^{2}+{\hat{\delta }}_{e}^{2}}$$

Where ${\hat{h}}^{2}$ represents estimates of heritability. Also, ${\hat{\delta }}_{a}^{2}$,$\;{\hat{\delta }}_{PE}^{2}$, ${\hat{\delta }}_{HYS}^{2}$, and ${\hat{\delta }}_{e}^{2}$ are the variance components of genetic effects, permanent environment, HYS, and residual variance, respectively. The residual effect was assumed to be distributed homogeneously in all models. The following formula was used to estimate each animal's (G)EBV at different survival times.

(5) $$\left(G\right)\text{EB}V_{mt}={ɸ}_{t}{\hat{a}}_{m}$$

$${ɸ}_{t}=\left(\begin{array}{c} {ɸ}_{t1}\\ {ɸ}_{t2}\\ {ɸ}_{t3}\\ \vdots \\ {ɸ}_{t100} \end{array}\right);\;{\hat{a}}_{m}=\left(\begin{array}{c} a_{m1}\\ a_{m2}\\ a_{m3}\\ \vdots \\ a_{m100} \end{array}\right)$$

The $\left(G\right)\text{EB}V_{mt}$ is the (G)EBV of animal m at time t, and ${ɸ}_{t}$ is the time vector related to time t. The respective $\left(G\right)\text{EB}V_{mt}$ values were added together with formula 6.

(6) $$\left(G\right)\text{EB}V_{m}=\sum_{t=1}^{100}\;\left(G\right)\text{EB}V_{mt}$$

Where $\left(G\right)\text{EB}V_{m}$ is the total breeding value for animal m and $\;\left(G\right)\text{EB}V_{mt}$ is the (G)EBV of animal m at time t.

### Validation

For this process two datasets of whole (W) and partial (P) along with a separate a group of test individuals, were used. The whole dataset included all phenotypes, while the partial dataset comprised phenotypes recorded up to a specific date. The test individuals were a group of young bulls selected at a specific time, according to detailed initial information. Subscript W denotes objects from the whole dataset, while subscript P denotes objects from the partial dataset. A total of 1,026 genotyped sires were identified, each with at least 40 daughters in 10 herds. To construct the partial dataset, 25% of the genotyped young sires were selected from the whole dataset based on their year of birth, and the records of their daughters were removed (Mäntysaari et al., 2010). The test group consisted of 290 sires born in years 2013-2016. Validation analyses were conducted to evaluate both the accuracy and dispersion of (G)EBV estimation. Accuracy was assessed using the Pearson correlation coefficient between (G)EBVs from the whole and partial datasets:

$Accuracy=\frac{Cov(G_{i\;W},G_{i\;P})}{{\left(Var_{G_{i\;W}}*\;Var_{G_{i\;P}}\right)}^{0.5}}$ (7) Where $G_{iW}$ and $G_{iP}$, are EBV or GEBV of test individuals in the whole and partial datasets, respectively, within the validation population for each method.

Dispersion of predictions was evaluated using the regression coefficient of $G_{i\;W}$ on $G_{i\;P}$:

$b_{W,P}=\frac{Cov(G_{i\;W},G_{i\;P})}{Var_{G_{i\;P}}}$ (8) High accuracy values and regression coefficients close to unity indicate reliable and unbiased (G)EBV predictions (Pahlavan et al., 2023). The intended evaluations should achieve higher accuracy and minimal dispersion ($b_{W,P}$). All analyses were performed using BLUPF90 software (Misztal et al., 2002).

## Results

**Fig. 1:**
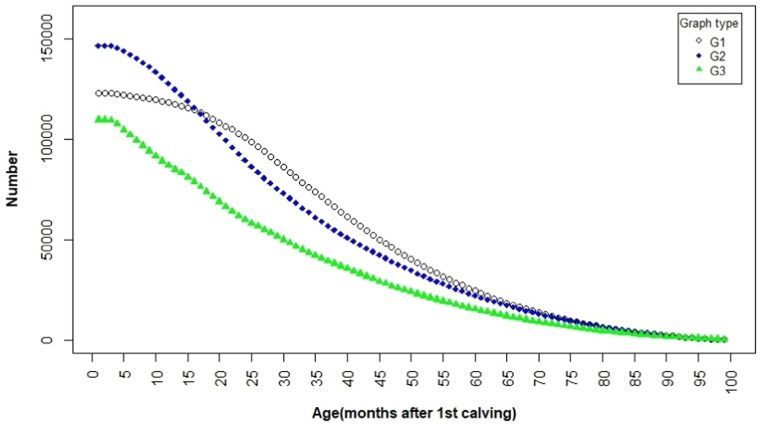
The survival up to 100 months after the first calving for three production groups of G1-Less than 7600 kg, G2-7600-10000 kg and G3-more than 10000 kg.

The average of survival of the cows was 36.2 (±21.9) months. Also, the average number of calvings were 2.7 (±1.5). In a research the mean of survival of Holstein cows in Iran was reported to be 34.0 months (Nejad et al., 2021). The average number of calvings of Holstein cows in the United States is 2.8 (Tsuruta et al., 2005), while in Serbia, it is 3.04 (Stanojević et al., 2016). The average survival of cows in G1, G2, and G3 groups were 31.9 (±22.6), 34.4 (±21.9), and 42.2 (±20.1) months, respectively (Fig. 1). The mean breeding value of survival for G1, G2, and G3 were 0.45, 0.30, and 0.41, respectively. The average cumulative survival breeding value for the three groups was 0.38. Additionally, the rank correlation between the breeding values of survival and the breeding value of 305-day milk production for first calving was -0.13 (*p* < 0.001). The rank correlations between the breeding value of survival and the breeding value of 305-day milk production for G1, G2, and G3 were 0.02 (*p* < 0.001), -0.18 (*p* < 0.001), and -0.21 (*p* < 0.001), respectively (Table 2). For each reported Spearman correlation, the standard error (SE), 99% confidence interval (CI), and two-tailed P-value were calculated. The SE was computed as $SE_{Z}=1/\sqrt{n-3}$ based on Fisher's Z transformation, the 99% CI was obtained by converting the Z interval back to the Spearman r scale, and the P-value was computed using the Z statistic. The resulting SE, CI, and P-values are presented in Table 2.

**Table 2:** Phenotypic and genetic information of uncensored cows for three production groups.

Yield^1^	Number^2^	survival^3^	calving^4^	Fixed^5^	EBV^6^	EBV^7^	correlation^7^	SE^8^	CI^9^	p-value
G1	109,602	31.9 (±22.6)	2.5 (±1.6)	0.05	0.45 (±1.58)	494 (±478)	0.02	0.00302	0.012,0.028	p < 0.001
G2	146,643	34.4 (±21.9)	2.5 (±1.6)	0.09	0.30 (±1.62)	753 (±438)	-0.18	0.00261	-0.189,-0.175	p < 0.001
G3	122,739	42.2 (±20.1)	3.0 (±1.4)	0.14	0.41 (±1.65)	975 (±456)	-0.21	0.00286	-0.221,-0.206	p < 0.001
Total	378,984	36.2 (±21.9)	2.7 (±1.5)	-	0.38 (±1.62)	750 (±493)	-0.13	0.00162	-0.134,-0.126	p < 0.001

1 The cows allocated to three groups according to the 305d-2x milk yield for the first calving, G1- Less than 7600 kg, G2- 7600-10000 kg and G3 -more than 10000 kg;

2 Number of cows;

3 Average of survival (month);

4 Average number of calving;

5 Fixed effect estimated;

6 Estimated Breeding Value for survival;

7 Estimated Breeding Value of the 305d-2x milk yield for the first calvin;

8 The rank correlation of survival Estimated Breeding Value with the breeding value of milk yield 305d-2x for the first calving;

9 The standard error; ^9^Confidence interval of 99%.

### Heritability

**Fig. 2:**
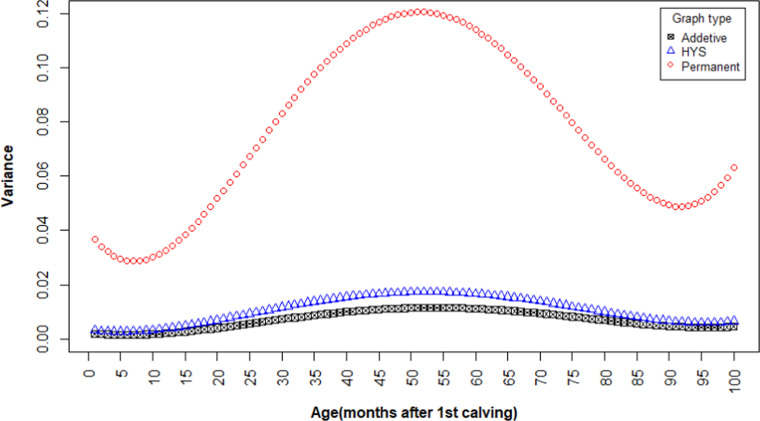
Permanent environmental effects, additive genetic variance, and herd-year- season variance of survival.

**Fig. 3:**
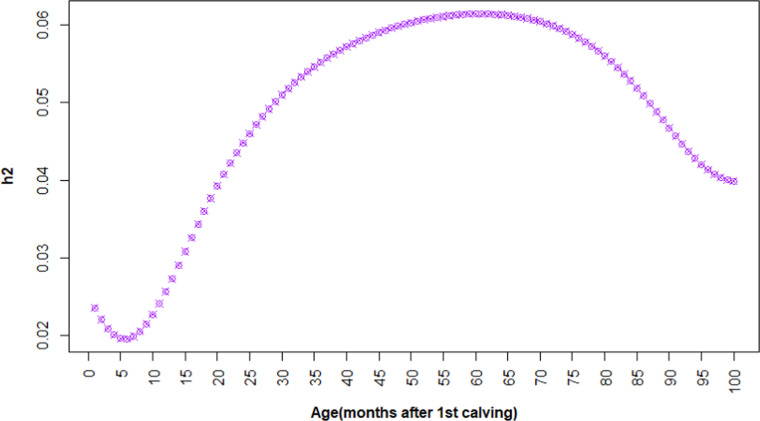
Heritability of Survival.

The variance components for survival were estimated using the RR method. The permanent environmental variance was the largest, while additive genetic variance was the smallest (Fig. 2). The heritability of the survival trait was 0.049, indicating a relatively small genetic contribution to the variation in this trait. Heritability gradually increased from around 0.02 at the beginning of the period to a peak of 0.06 at approximately 60 months after first calving, and then declined slightly up to 100 months (Fig. 3).

### Accuracy of prediction

**Fig. 4:**
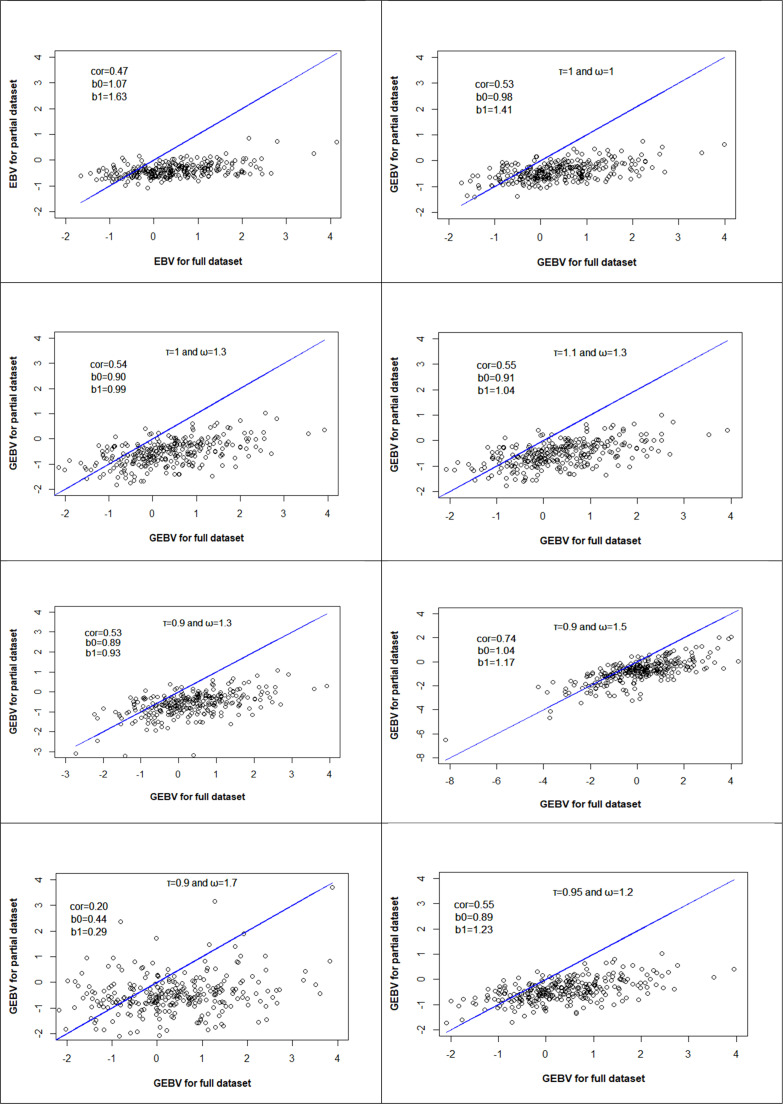
Accuracy (cor), intercept (b_0_), and dispersion of predictions (b_1_) of EBV or GEBV for the test individual used in the validation process. Different values for τ and ω were tested in the ssGBLUP model. The first graph shows the results obtained from the standard BLUP analysis.

Using both of genomic and pedigree data in the ssGBLUP model increased prediction accuracy and reduced dispersion compared to the traditional BLUP model. The prediction accuracy was 0.47 (*p* < 0.001) for the BLUP model and increased to 0.53 (*p* < 0.001) for the ssGBLUP model when the default values of τ = ω = 1 were used. Corresponding dispersion values were 1.63 and 1.41, respectively. The ssGBLUP method also showed reduced bias, with the intercept in the regression analysis being closer to zero compared to BLUP. Furthermore, using scaling factors of τ = 0.9 and ω = 1.5 in the ssGBLUP model improved prediction accuracy to 0.74 (*p* < 0.001) and decreased dispersion to 1.16. These represent respective improvements of 0.27 and 0.47 in accuracy and dispersion compared to the BLUP model, and 0.21 and 0.24 compared to the default ssGBLUP model. Different combinations of τ and ω values were tested in validation process. For each reported Pearson correlation, the standard error (SE), 99% confidence interval (CI), and two-tailed P-value were calculated. The SE was computed as $SE_{Z}=1/\sqrt{n-3}$ based on Fisher's Z transformation, the 99% CI was obtained by converting the Z interval back to the Pearson r scale, and the P-value was computed using the Z statistic. The resulting SE, 99% CI, and P-values are presented in Table 3. The changes in prediction accuracy and dispersion in those combinations are shown in Fig. 4.

**Table 3:** Accuracy, intercept (b_0_) and regression coefficients (b_1_) for survival trait in RR-BLUP and RR-ssGBLUP Models.

Model	n	τ	ω	b_0_	b_1_	Accuracy	SE	CI	p-value
RR-BLUP	290	-	-	1.07	1.63	0.47	0.059	0.343,0.579	p < 0.001
RR-ssGBLUP	290	1	1	0.98	1.41	0.53	0.059	0.411,0.630	p < 0.001
RR-ssGBLUP	290	1	1.3	0.90	0.99	0.54	0.059	0.425,0.639	p < 0.001
RR-ssGBLUP	290	1.1	1.3	0.91	1.04	0.55	0.059	0.439,0.646	p < 0.001
RR-ssGBLUP	290	0.9	1.3	0.89	0.93	0.53	0.059	0.411,0.630	p < 0.001
RR-ssGBLUP	290	0.9	1.5	1.04	1.17	0.74	0.059	0.664,0.802	p < 0.001
RR-ssGBLUP	290	0.9	1.7	0.44	0.29	0.20	0.059	0.050,0.340	p < 0.001
RR-ssGBLUP	290	0.95	1.2	0.89	1.23	0.55	0.059	0.439,0.646	p < 0.001

n is sample size; τ, ω are the optimal scaling factors; b_0_ is intercept; b_1_ is regression coefficient or dispersion of predictions; Accuracy was assessed using the Pearson correlation coefficient between (G)EBVs from the whole and partial datasets; SE is the standard error for accuracy; CI is confidence interval of 99% for accuracy.

## Discussion

High-producing cows had lower breeding values for survival than low-producing cows, yet they remained longer in the herd. The results suggest that non-genetic or environmental factors may contribute to the longer survival of high-producing cows. Breeders tend to retain high-producing cows in the herd and may invest more in their care (including treatment and reproduction) compared to low- and medium-producing cows. The results of a study showed the relative risk of culling was 3.6 for the low-producing group and 0.41 for the high-producing group, indicating that the risk of culling in the low-producing group was nine times higher (Kern et al., 2016). Cows with higher milk production and longer survival reduce replacement costs, increase lifetime yield, and improve the overall profitability of dairy farms. Raising high-producing animals longer in the herd not only increase return of investment but also reduces economic losses associated with early culling, making survival an economically significant trait for selection.

The survival of high-producing Brazilian Holstein cows is 41 months, while survival of low-producing cows is about 38 months (Vernaschi et al., 2022). Similarly, higher-producing animals of Canadian Ayrshire and Jersey breeds have longer survival (Galbraith, 2003). These differences may be the result of differences in management practices, environmental conditions, and breeding strategies. Adjusting for within-herd production levels tends to reduce these differences when estimating breeding values using random regression models (Van Pelt et al., 2018). Such international comparisons emphasize the importance of both genetic and non-genetic factors in improving survival in dairy cattle.

The rank correlations between the breeding values for survival and 305-day milk production varied across production groups. In low-producing cows (G1), the correlation was slightly positive (0.02), indicating almost non-genetic relationship between survival and milk yield in this group. In contrast, moderate- and high-producing cows (G2 and G3) showed negative correlations (-0.18 and -0.21, respectively), suggesting that higher milk production may be associated with lower breeding values for survival. However, the observed survival of high-producing cows may also be influenced by non-genetic or environmental factors, which could reduce the negative genetic association.

The heritability of survival estimated in this study was 0.049, indicating a relatively low genetic variance for this trait. The low heritability shows that most of the variation in cow survival is due to environmental factors. Therefore, genetic improvement of survival is slow and requires large data and precise selection of animals.

In the early months after first calving, heritability was low (around 2% at month 6), likely because most related animals were still alive and managed within the same herds, reducing observable genetic differences in survival. As cows were culled or died over time, genetic differences became more pronounced, causing heritability to increase up to month 61. After this point, with more family members culled, differences between families diminished again, leading to a decrease in heritability.

Comparisons with other studies show that the heritability of survival in Iranian Holstein cows ranges between 0.012-0.058 (Ashrafian et al., 2023), while for Dutch Holstein cows it ranges between 0.02-0.031 (Van Pelt et al., 2015). The heritability estimate in this study is slightly higher than those reported in other studies, which may be attributable to differences in environmental conditions, management practices, or the data sets used.

Nevertheless, even low heritability allows for genetic improvement, especially when advanced models and genomic information are incorporated. Additionally, understanding non-genetic factors and improving management practices can complement efforts for genetic selection improvement.

Therefore, the low heritability of survival highlights the importance of combining management strategies with genetic improvement to enhance survival in dairy herds.

The findings indicate that using genomic data in genetic evaluation through the ssGBLUP model significantly improves prediction accuracy (Fig. 4). The improved accuracy and reduction in dispersion demonstrate clear advantages of this method compared to traditional BLUP. In particular, tuning the scaling factors in the H matrix (i.e., τ and ω) played a key role in optimizing model performance. These results is in agreement with other research (Afrazandeh et al., 2022) showing that scaling factors can increase prediction accuracy and reduce dispersion. Moreover, the correct selection of τ and ω values ensures that the estimated genetic values for animals in the partial dataset more closely match those in the full dataset. Also, it shows that selection of these scaling factors reduces deviations from the expected regression line and provides more accurate and less biased estimates.

In this research the survival of Iranian Holstein cattle was studied with a large dataset including genomic information. A random regression model was applied to evaluate additive genetic and permanent environmental effects in months after first calving. The ssGBLUP model significantly outperformed the traditional BLUP approach, particularly when optimized scaling factors (τ = 0.9, ω = 1.5) were used. These results support the use of genomic and pedigree data to improve the accuracy and reduce the bias of survival predictions. The higher survival observed in high-producing cows appears to be influenced by environmental factors underscoring the combined role of genetic and non-genetic factors. Selection of high-producing dairy cows which are daughters of sires with high survival genetic potential can improve more profitable and sustainable dairy systems.

This study presents several unique aspects that distinguish it from previous research. First, it utilizes a very large national dataset comprising 1.36 million Iranian Holstein cows across 4,370 herds, representing one of the most extensive survival datasets analyzed in the region. Such a large-scale dataset enables robust estimation of genetic parameters with high precision, which few studies in developing dairy systems have achieved. Second, the regional novelty and applied importance of this study are noteworthy: genomic evaluation of survival traits has rarely been conducted in Iranian Holstein cattle. This work represents a step toward developing a genomic selection system for national dairy improvement programs, directly addressing the practical needs of Iranian animal breeding. Third, the methodological integration is a major strength: applying random regression (RR) within the single-step genomic BLUP (ssGBLUP) framework allows the simultaneous estimation of time-dependent genetic parameters while incorporating genomic information. This integration enhances the predictive accuracy and provides a more comprehensive approach to evaluating survival traits in a longitudinal context.

## Ethics approval

The data used in this study were obtained from previously collected records; therefore, ethics approval was not required.

## Data and model availability statement

The data used in this study were obtained from the Animal Breeding Center of Iran. These data are not publicly available. Access to the data may be possible by contacting the data provider and explaining the purpose of the request.

## Declaration of generative AI and AI-assisted technologies in the writing process

During the preparation of this work the author(s) did not use any AI and AI-assisted technologies.

## Financial support statement

This research received no financial support

## CRediT authorship contribution statement

**M. Afrazandeh:** Writing – review & editing, Writing – original draft, Visualization, Validation, Software, Resources, Methodology, Investigation, Formal analysis, Data curation, Conceptualization. **J.J. Tourchi:** Visualization. **A. Kazemi:** Visualization, Resources, Methodology, Data curation.

## Declaration of competing interest

The authors declare that they have no known competing financial interests or personal relationships that could have appeared to influence the work reported in this paper.
